# The Lateral Occipito-temporal Cortex Is Involved in the Mental Manipulation of Body Part Imagery

**DOI:** 10.3389/fnhum.2017.00181

**Published:** 2017-04-11

**Authors:** Mitsuru Kikuchi, Tetsuya Takahashi, Tetsu Hirosawa, Yumi Oboshi, Etsuji Yoshikawa, Yoshio Minabe, Yasuomi Ouchi

**Affiliations:** ^1^Research Center for Child Mental Development, Graduate School of Medical Science, Kanazawa UniversityKanazawa, Japan; ^2^Department of Psychiatry and Neurobiology, Graduate School of Medical Science, Kanazawa UniversityKanazawa, Japan; ^3^Department of Biofunctional Imaging, Medical Photonics Research Center, Hamamatsu University School of MedicineHamamatsu, Japan; ^4^Central Research Laboratory, Hamamatsu Photonics K.K.Hamamatsu, Japan

**Keywords:** extrastriate body area, hand imagery, lateral occipito-temporal cortex, mental rotation, transcranial direct current stimulation

## Abstract

The lateral occipito-temporal cortex (LOTC), including the extrastriate body area, is known to be involved in the perception of body parts. Although still controversial, recent studies have demonstrated the role of the LOTC in higher-level body-related cognition in humans. This study consisted of two experiments (E1 and E2). The first (E1) was an exploratory experiment to find the neural correlate of the mental manipulation of body part imagery, in which brain cerebral glucose metabolic rates and the performance of mental rotation of the hand were measured in 100 subjects who exhibited a range of symptoms of cognitive decline. In E1, we found that the level of glucose metabolism in the right LOTC was significantly correlated with performance in a task involving mental manipulation of the hand. Next, in E2, we performed a randomized, double-blind, controlled intervention study (clinical trial number: UMIN 000018310) in younger healthy adults to test whether right occipital (corresponding to the right LOTC) anodal stimulation using transcranial direct current stimulation (tDCS) could enhance the mental manipulation of the hand. In E2, we demonstrated a significant effect of tDCS on the accuracy rate in a task involving mental manipulation of the hand. Although further study is necessary to answer the question of whether these results are specific for the mental manipulation of body parts but not non-body parts, E1 demonstrated a possible role of the LOTC in carrying out the body mental manipulation task in patients with dementia, and E2 suggested the possible effect of tDCS on this task in healthy subjects.

## Introduction

Previous neuroimaging studies have identified two brain regions that are more sensitive to visually perceived body parts than to non-human objects in the visual cortex. These regions are known as the extrastriate body area (EBA), which is located in the lateral occipito-temporal cortex (LOTC) (Downing et al., 2001), and the fusiform body area (FBA), which is found ventrally in the fusiform gyrus (Peelen and Downing, 2005; Schwarzlose et al., 2005). Since their identification, the functions of the EBA and FBA in higher-level body-related cognition have been elucidated through correlational evidence in human brain imaging studies (Peelen and Downing, 2007; Taylor et al., 2007; Moro et al., 2008; Ferri et al., 2012b; Kitada et al., 2014). Furthermore, as causal evidence, transcranial magnetic stimulation studies (Peelen and Downing, 2007; Urgesi et al., 2007a,b; Pitcher et al., 2012) and a brain lesion study (Moro et al., 2008) also indicated that the EBA and the FBA are responsible for the representation of body part identification. In addition to the EBA’s role in recognition, the EBA has also been reported to be active when individuals perform movements, prepare self-actions, or perceive the body movements of others (Astafiev et al., 2004; David et al., 2007; Peelen and Downing, 2007; Urgesi et al., 2007a; Ishizu et al., 2009; Orlov et al., 2010; Kuhn et al., 2011; van Nuenen et al., 2012). These findings suggest that the EBA not only receives sensory inputs regarding others’ body information but also represents the human body in a dynamic manner, including kinesthetic feedback for one’s own actions. If the EBA receives kinesthetic information about self-actions, it is easy to speculate that the EBA would also be activated during the manipulation of body imagery.

The mental rotation task is a well-established paradigm to study the cognitive process of mentally rotating objects. The brain network that governs mental rotation has been studied using a variety of stimuli (e.g., hands) (Bonda et al., 1995; Parsons et al., 1995; Kosslyn et al., 1998; Vingerhoets et al., 2002; Wraga et al., 2003; de Lange et al., 2005; Seurinck et al., 2005; de Lange et al., 2006; Creem-Regehr et al., 2007; Corradi-Dell’Acqua et al., 2009; Ferri et al., 2012a; Papeo et al., 2012). Using activation likelihood estimation meta-analysis, a recent study (Tomasino and Gremese, 2015) showed activation by the mental rotation task itself in the bilateral inferior and superior parietal lobule, the precentral gyrus, the inferior frontal gyrus, the middle frontal gyrus, the supplementary motor area, the insula, the inferior and middle occipital gyrus and the cerebellum. They also demonstrated that the mental rotation of bodily parts activates the cerebellum, the middle and inferior occipital and calcarine gyrus, the superior parietal lobule, the bilateral postcentral gyrus, the left postcentral gyrus, the left inferior parietal lobe and the right supramarginal gyrus, the left precentral gyrus and the bilateral inferior frontal gyrus, the left superior frontal gyrus, the right middle frontal gyrus and the medial posterior frontal gyrus in addition to the right insula. Therefore, not only motor areas but also visual areas, including the EBA and FBA, seem to be involved in the mental rotation task.

**FIGURE 1:**
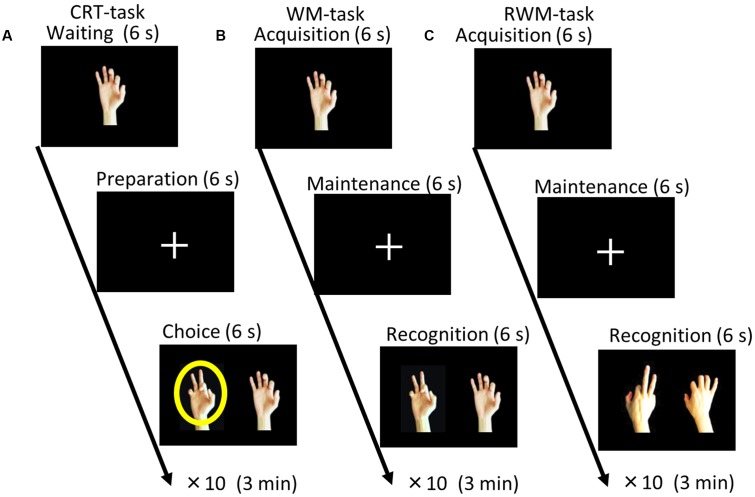
**Task paradigms. (A)** Choice reaction time (CRT) task. The participants were instructed to select the right or left picture that included a circle (i.e., a target) by pressing a button with their right or left hand as soon as possible after a circle appeared on the screen. **(B)** During the recognition phase of the simple visual working memory task (WM task), the participants were instructed to select the picture that depicted the same hand as that presented during the acquisition phase (non-flipped images). **(C)** During the recognition phase of the visual working memory with mental rotation task (RWM task), the participants were instructed to select the picture that depicted the same hand, which was flipped from the palm side (acquisition) to the back side (recognition). Each condition consisted of 10 trials with different hand shape pictures and lasted for a duration of 3 min.

With the aid of decompensated brains (i.e., elderly subjects mainly consisting of patients with dementia; *n* = 100), the first part of the present study (E1) intended to examine the brain region that is critical in cognitive decline (i.e., decompensation) in the mental rotation task (**Figure 1**). From the results of E1, we discovered that lower glucose metabolic ratios in the EBA and the FBA were correlated with lower performance on the mental manipulation of the hand imagery task.

Transcranial direct current stimulation (tDCS) is a non-invasive stimulation method that can induce prolonged excitability changes in the cortex, as shown in several studies in humans (Nitsche and Paulus, 2000, 2011; Nitsche et al., 2003, 2007, 2008; Antal et al., 2004a,b; Labruna et al., 2016). In tDCS, weak currents are applied to the cortex via two electrodes placed on the scalp. In the visual cortex, tDCS modulated the amplitude of visual-evoked potentials in a polarity-dependent way: anodal stimulation increased the amplitude of the visual-evoked potentials, but cathodal stimulation decreased it (Antal et al., 2004a). Based on the results of E1, we hypothesized that active stimulation of the LOTC using tDCS would enhance performance on the mental manipulation of the hand imagery task. In the second experiment (E2), we used the tDCS technique to perform a randomized, double-blind, controlled study to test this hypothesis.

## Materials and Methods

### Experiment 1: Explorative Brain Imaging Study

#### Participants

We examined 100 elderly subjects, including 22 healthy elderly subjects (9 men and 13 women; mean age 68.0 ± 7.28 years), 52 patients diagnosed with Alzheimer’s disease (AD) (30 men and 22 women; mean age 67.4 ± 8.63 years), 14 patients diagnosed with fronto-temporal dementia (FTD) (6 men and 8 women; mean age 64.3 ± 6.73 years), and 12 patients diagnosed with mild cognitive impairment (MCI) (6 men and 6 women; mean age 59.8 ± 6.69 years). Therefore, our subjects consisted of 22 healthy and 78 cognitively impaired (from MCI to dementia) elderly subjects. All patients with dementia and MCI were diagnosed on the basis of an extensive clinical history and physical examinations. The diagnoses of AD, FTD, and MCI were based on the criteria of the National Institute of Neurological and Communicative Disorders and Stroke-Alzheimer’s Disease and Related Disorders Association (NINCDS/ADRDA) (McKhann et al., 1984), the Diagnostic and Statistical Manual of Mental Disorders-IV (DSM-IV) (American Psychiatric Association, 1994), the Lund and Manchester criteria (Neary et al., 1998), and the Petersen MCI criteria (Petersen, 2004).

All participants underwent brain MRI and [^18^F] fluoro-deoxyglucose (FDG)-PET scans. The present study was approved by the Ethics Committee of Hamamatsu Medical Center (Heisei 8-1), and written informed consent was obtained from all participants prior to enrollment. The methods were carried out in accordance with approved guidelines.

#### Working Memory Tasks

Three visual tasks were presented on a liquid crystal screen in front of the subjects. As shown in **Figure 1**, one choice reaction time task (CRT task) and two types of working memory tasks were employed (Kikuchi et al., 2011). In the CRT task, the participants were instructed to respond to a circle (i.e., a target) that appeared on the screen by pressing a button with the hand that was located on the same side as the circle (either their right or their left hand) as soon as possible. One working memory task consisted of a simple visual working memory task (WM task) that involved pictures of various hand shapes (palm side only) and the other working memory task consisted of a visual working memory task with mental rotation (RWM task), which used pictures of various hand shapes (palm and back side).

#### Inverse Efficiency (IE) Scores for the CRT, WM and RWM Tasks

To control for speed–accuracy trade-offs in the cognitive outcome data, we calculated a value [inverse efficiency (IE) score (Townsend and Ashby, 1983; Ludwig et al., 2011)] by dividing the median response time (RT) by the accuracy rate in each task condition. Because the RT was measured in ms and was divided by a unitless number, the IE score was expressed in ms as well. For instance, an average RT of 1000 ms and a 10% error rate would yield an IE value of 1111 ms (1000/(1–0.1)). A lower IE score indicates better performance.

#### MRI Scanning

All participants underwent 3-dimensional MRI immediately before the PET measurements. During this process, a static magnet (0.3 T MRP7000AD; Hitachi, Tokyo, Japan) was used in the 3-dimensional mode (Ouchi et al., 2001).

#### PET Scanning and Image Data Acquisition

The patients underwent a series of PET measurements after completing the battery of neuropsychological tests and the MRI examination. A high-resolution brain PET scanner was used (SHR12000; Hamamatsu Photonics K.K., Hamamatsu, Japan) (Ouchi et al., 1999). After a subject’s head was fixed with a thermoplastic face mask and a 10-min transmission scan was acquired, a static 15-min PET scan was performed 45 min after an injection of a 1.2 MBq/kg dose of [^18^F] FDG.

#### Image Data Processing

To evaluate glucose metabolism, a semiquantitative ratio index of [^18^F]FDG was calculated to obtain the standardized uptake value ratio (SUVR) (Ouchi et al., 2009).

#### Voxel-Wise Statistical Analysis

SPM8 was used for the voxel-wise analysis (voxel-size; 2 mm × 2 mm × 2 mm resolution). All [^18^F]FDG-SUVR parametric images were first normalized to the MNI space and smoothed with an 8 mm isotropic Gaussian kernel. Voxel-based correlations were computed between [^18^F]FDG-SUVR parametric images and IE scores or accuracy rates in the three conditions using a multiple regression model with the statistical threshold set at *p* = 0.05 (corrected with FWE) for the peak height. These analyses were applied for the data from the healthy elderly subjects (*n* = 22) and all participants, including the cognitively impaired subjects (*n* = 100).

### Experiment 2: tDCS Study

#### Study Design and Setting

Based on the results from the first part of the present study, we conducted a second randomized, double-blind, controlled study. The study was registered with the University Hospital Medical Information Network (UMIN) Clinical Trials Registry (number UMIN000018310). Forty healthy men (age range, 20–43 years; all right-handed as assessed by the Edinburgh Handedness Inventory) performed the same tasks as described in the current PET study while receiving anodal tDCS to either the right occipital cortex covering the LOTC (i.e., the region highlighted in the mental rotation task) or the right dorsolateral prefrontal cortex (DLPFC) (i.e., the region used for the control condition) in combination with cathodal tDCS of the right DLPFC or the right occipital cortex (the former condition was referred to as occipital anodal/frontal cathodal stimulation and the latter condition was referred to as frontal anodal/occipital cathodal stimulation). The control condition involved sham tDCS. The participants were not taking any medications, had no history of neurological or psychiatric disease, and had normal physical and neurological examinations. All subjects were naive to tDCS. Written informed consent was obtained prior to participation in the study. The Ethics Committee of Kanazawa University Hospital approved the methods and procedures and the methods were carried out in accordance with approved guidelines. The demographic data for all participants are presented in Supplementary Table S1.

**FIGURE 2:**
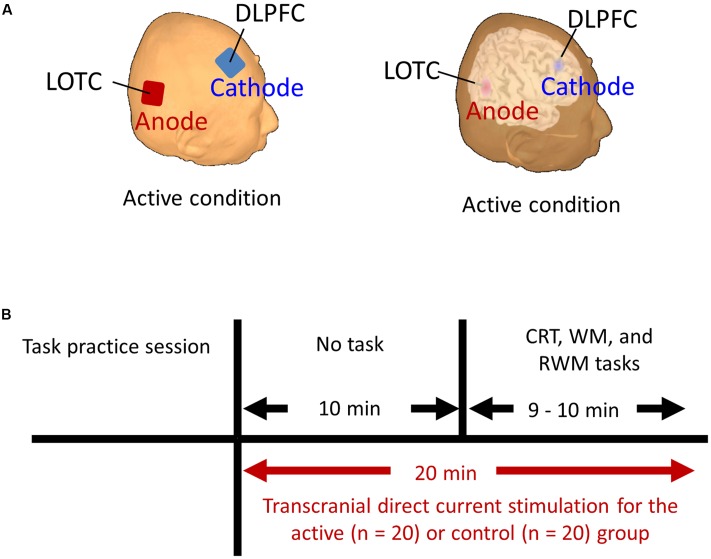
**(A)** Stimulation targets (i.e., LOTC and DLPFC] marked on the reconstructed scalp and brain surface of one subject in the active condition. **(B)** Schematic representation of the experimental design. Each participant began to perform the CRT, WM, and RWM tasks after receiving active or control stimulation for 10 min. The stimulation continued throughout the duration of the three tasks and for an additional 10 min. CRT, choice reaction time. WM, working memory. RWM, working memory with hand mental rotation. LOTC, lateral occipito-temporal cortex. DLPFC, dorsolateral prefrontal cortex.

Before the test, each subject underwent a practice session to ensure that they understood and were well-trained for the tasks used in the present study (i.e., the CRT, WM, and RWM tasks shown in **Figure 1**). A direct current was induced by two saline-soaked surface sponge electrodes (35 cm^2^) and delivered by a battery-driven, constant current stimulator (DC-STIMULATOR Plus, neuroConn GmbH, Germany). The participants were randomly assigned to receive occipital anodal/frontal cathodal (*n* = 20; active group), frontal anodal/occipital cathodal (*n* = 10; control group 1), or sham tDCS (*n* = 10; control group 2). During stimulation in the occipital anodal/frontal cathodal condition, as shown in **Figure 2A**, the anode electrode was placed over PO8 (international EEG 10/10 system) and the cathode electrode was placed over F4 (international EEG 10/10 system). During stimulation in the frontal anodal/occipital cathodal condition, the polarity was reversed. During active stimulation, the participants received a constant current with an intensity of 2 mA. tDCS was initiated 10 min before the task began and was delivered throughout the duration of the three tasks, which lasted approximately 10 min in total (**Figure 2B**). During sham stimulation, the electrodes were placed at the same positions used during active stimulation, but the stimulator was turned on for only 30 s. Therefore, the participants may have experienced a tDCS-induced itching sensation at the beginning of the session but received no active current for the remaining stimulation period.

#### Data Analysis of the tDCS Study

To determine whether anodal stimulation to the right occipital region affected the mental manipulation of body part imagery, we evaluated the performance of the subjects (IE score and accuracy) during the CRT, WM, and RWM tasks. The performances of the subjects in the active group (i.e., occipital anodal/frontal cathodal stimulation) were compared with those of the subjects in the control group (i.e., a mixture of frontal anodal/occipital cathodal stimulation and sham stimulation, *n* = 20). Based on the results of E1 in the present study, for the RWM task, we hypothesized that the IE score will be lower and the accuracy rate will be higher during stimulation in the occipital anodal/frontal cathodal condition compared with the control condition.

For the IE score of the RWM task, an unpaired *t*-test (one-sided) was used to compare the two groups (i.e., active vs. control stimulation). For the accuracy rate of the RWM task, a Wilcoxon rank-sum test (one-sided) was used to compare the two groups (i.e., active vs. control stimulation). To avoid the risk of low statistical power caused by the smaller sample size, we added complementary analyses between sub-conditions (i.e., we divided the control condition into two conditions) to test the difference between the active (occipital anodal/frontal cathodal stimulation; *n* = 20) and the reversed (frontal anodal/occipital cathodal stimulation; *n* = 10) or the sham conditions (*n* = 10).

As complementary analyses, a two-way ANOVA was performed (task × tDCS condition) for the IE score of the three tasks. The within-subjects factor was the task effect (CRT vs. WM vs. RWM tasks) and the between-subjects factor was the tDCS effect (active vs. control condition or active vs. 2 control conditions).

The significance level was set at 0.05.

## Results

### Experiment 1: Explorative Brain Imaging Study

As shown in Supplementary Figure S1 and **Table 1**, the healthy elderly tended to show higher accuracy, shorter RTs, and lower IE scores compared with the cognitively impaired patients in all tasks. The diversified data of the cognitive and brain metabolic profiles in the 22 healthy and 78 cognitively impaired subjects allowed us to conduct correlation analyses.

**Table 1:** Demographic characteristics of all subjects in the PET study.

	All subjects Mean (*SD*)	Healthy subjects Mean (*SD*)	Cognitively impaired subjects Mean (*SD*)
Total number	100	22	78
Accuracy score for CRT task	9.6 (1.0)	9.9 (0.4)	9.5 (1.1)
Accuracy score for WM task	8.4 (1.9)	9.2 (1.0)	8.2 (2.1)
Accuracy score for RWM task	8.7 (1.7)	9.6 (0.6)	8.4 (1.8)
Reaction time for CRT task (ms)	1048 (751)	673 (245)	1154 (811)
Reaction time for WM task (ms)	1663 (931)	1031 (237)	1841 (976)
Reaction time for RWM task (ms)	1941 (1055)	1167 (289)	2125 (1097)
Inverse efficiency score for CRT task (ms)	1170 (1081)	682 (245)	1308 (1183)
Inverse efficiency score for WM task (ms)	2341 (2081)	1118 (215)	2686 (2238)
Inverse efficiency score for RWM task (ms)	2537 (2324)	1206 (267)	2912 (2505)

*CRT, choice reaction time; WM, visual working memory; RWM, visual working memory with mental rotation condition; ms, milliseconds*.

**FIGURE 3:**
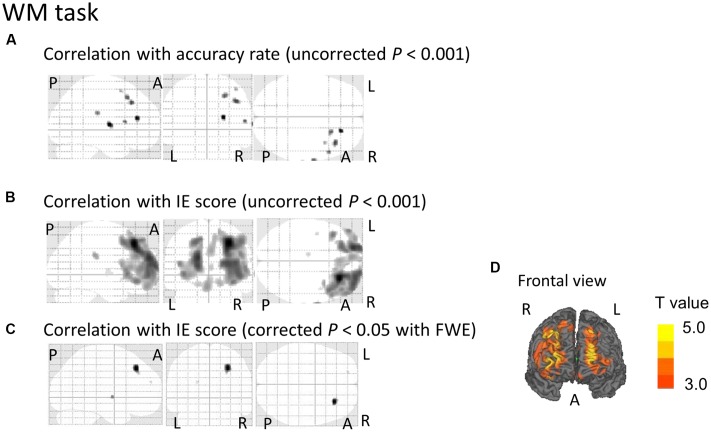
**SPM analyses with multiple regression models in which the accuracy rate (A)** or the inverse efficiency (IE) scores **(B–D)** for the WM conditions were used as independent variables. **(A)** A decrease in the [^18^F]FDG-SUVR in the right frontal cortices was associated with poorer performance (i.e., lower accuracy rate) in the WM task. However, this significant association disappeared if we employed a conservative statistical threshold set at *p* = 0.05 (corrected with FWE). **(B,D)** A decrease in the [^18^F]FDG-SUVR in the frontal cortices was associated with poorer performance (i.e., higher IE) in the WM task. **(C)** This significant correlation was still observed in the right frontal cortex if we employed a conservative statistical threshold set at *p* = 0.05 (corrected with FWE). The yellow color bar indicates the *T*-value. L, left hemisphere. R, right hemisphere. A, anterior. P, posterior.

**FIGURE 4:**
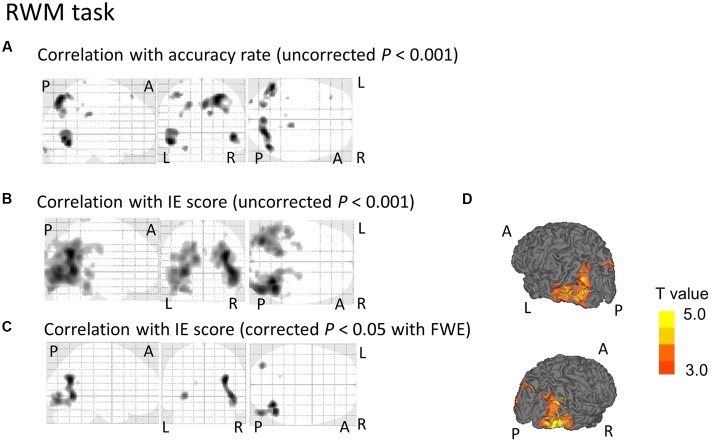
**SPM analyses with multiple regression models in which the accuracy rate (A)** or the IE scores **(B–D)** for the RWM conditions were used as independent variables. **(A)** A decrease in the [^18^F]FDG-SUVR in the occipital and parietal cortices was associated with poorer performance (i.e., lower accuracy rate) in the RWM task. However, this significant association disappeared if we employed a conservative statistical threshold set at *p* = 0.05 (corrected with FWE). **(B,D)** A decrease in the [^18^F]FDG-SUVR in the occipital and parietal cortices was associated with poorer performance (i.e., higher IE) in the RWM task. **(C)** This significant correlation was still observed in the bilateral occipito-temporal cortices if we employed a conservative statistical threshold set at *p* = 0.05 (corrected with FWE). The yellow color bar indicates the *T*-value. L, left hemisphere. R, right hemisphere. A, anterior. P, posterior.

Regarding the correlation between [^18^F]FDG-SUVR and the accuracy rates, with the conservative statistical threshold set at *p* = 0.05 (corrected with FWE), no significant correlation was found for the three tasks (i.e., the CRT task, the WM task and the RWM task) in healthy subjects (*n* = 22) or in all participants, including the cognitively impaired subjects (n = 100). At the risk of type I error, if we employed a statistical threshold set at *p* = 0.001 (uncorrected) for all subjects (*n* = 100), a decrease in the [^18^F]FDG-SUVR in the right frontal areas was associated with a lower accuracy rate in the WM task (**Figure 3A**) and a decrease in the [^18^F]FDG-SUVR in the bilateral occipital and parietal areas was associated with a lower accuracy rate on the RWM task (**Figure 4A**).

Regarding the correlation between [^18^F]FDG-SUVR and IE scores in the healthy elderly subjects (*n* = 22), with the conservative statistical threshold set at *p* = 0.05 (corrected with FWE), no significant correlation was found for the three tasks. In all participants, including the cognitively impaired subjects (*n* = 100), with the conservative statistical threshold set at *p* = 0.05 (corrected with FWE), no significant correlation was found for the CRT task. For the WM task, decreases in the [^18^F]FDG-SUVR in the right middle frontal gyrus (*z* score = 4.91, cluster size = 100 voxels) and the left superior frontal gyrus (*z* score = 4.46, cluster size = 4 voxels) were associated with lower performance (**Figures 3B–D** and **Table 2**). For the RWM task, which was the focus of our study, a decrease in the [^18^F]FDG-SUVR in the bilateral EBA, the right FBA (i.e., decreased glucose metabolism in the right middle/inferior occipital gyri and the middle temporal gyrus), and in the left middle occipital gyrus was significantly correlated with poorer task performance (**Figures 4B–D** and **Table 2**).

**Table 2:** Brain regions in which a decrease in the [^18^F]FDG-SUVR was significantly associated with poorer task performance.

		Values at peak voxel						
			Coordinate^†^					
Brain Areas^∗^		L/R	x	y	z	Z score (*T*-value)	*P*_FWE-corrected_ (*df*)	Cluster size (The number of voxels)
**Relative to the lower WM task performance**					
Frontal lobe	Middle frontal gyr.	R	28	32	46	4.91 (5.25)	0.007 (96)	100
	Superior frontal gyr	L	–18	56	24	4.46 (4.72)	0.046 (96)	4
**Relative to the lower RWM task performance**								
Occipital lobe	Middle occipital gyr	R	34	–62	24	5.54 (6.03)	<0.001 (96)	939^‡^
Temporal lobe	Middle temporal gyr	R	44	–58	0	5.39 (5.84)	0.001 (96)	^‡^
Occipital lobe	Inferior occipital gyr	R	48	–82	–8	5.17 (5.57)	0.002 (96)	^‡^
Occipital lobe	Middle occipital gyr	L	–32	–80	0	5.08 (5.45)	0.003 (96)	133

*SPM analyses with multiple regression models in which the IE scores in the three conditions were used as independent variables. ^∗^Statistical significance was assumed at an individual voxel level of *p* < 0.05, corrected (FWE). ^†^Montreal Neurological Institute (MNI) brain atlas coordinates: x = distance in millimeters to the right (+) or left (–) of the midline; y = distance anterior (+) or posterior (–) to the anterior commissure; z = distance superior (+) or inferior (–) to a horizontal plane through the anterior and posterior commissures. Gyr, gyrus. *df*, degrees of freedom. ^‡^Three peak voxels belonging to the same cluster*.

### Experiment 2: tDCS Study

**FIGURE 5:**
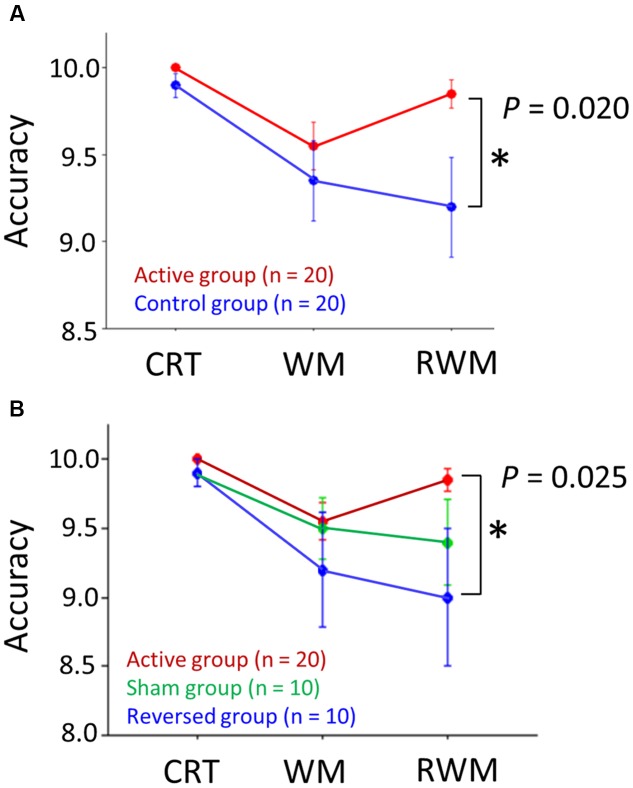
**(A)** Accuracy scores in the three tasks (i.e., CRT, WM, and RWM) with the application of active (red circles) and control (blue circles) transcranial direct current stimulation (tDCS). The RWM task scores during right occipital anodal stimulation (i.e., the active condition, *n* = 20) were significantly higher than those during the control stimulation (*n* = 20). **(B)** If the control condition was divided into two conditions, i.e., the reversed condition (frontal anodal/occipital cathodal stimulation; *n* = 10) (blue circles) and sham condition (*n* = 10) (green circles), the RWM task scores during the active condition (*n* = 20) were significantly higher than those during the reversed condition (*n* = 10) but not than those during the sham condition (*n* = 10). The error bar indicates 1 standard error. CRT, choice reaction time. WM, working memory. RWM, working memory with hand mental rotation.

Regarding the IE score, an unpaired *t*-test (one-sided) did not reveal significantly higher performance (i.e., a lower IE score) in the right occipital anodal stimulation group (i.e., active condition, *n* = 20) compared to the control group (*n* = 20) (*t* = 0.684, *p*-value > 0.05), which did not support our hypothesis. However, regarding the accuracy rate, as shown in **Figure 5A**, a Wilcoxon rank-sum test (one-sided) revealed a significantly higher performance (i.e., higher accuracy rate) in the RWM task in the right occipital anodal stimulation group (i.e., active condition, *n* = 20) than in the control group (*W* = 135.5, *p*-value = 0.020), which supported our hypothesis.

Next, we divided the control condition into two conditions, i.e., the reversed (frontal anodal/occipital cathodal stimulation; *n* = 10) condition and the sham condition (*n* = 10). Regarding the IE score, an unpaired *t*-test (one-sided) did not reveal a significantly higher performance (i.e., lower IE score) in the active condition group (*n* = 20) than in the reversed condition group (*n* = 10) (*t* = –0.388, *p*-value > 0.05) or the sham condition group (*n* = 10) (*t* = 1.643, *p*-value > 0.05). Regarding the accuracy rate, a Wilcoxon rank-sum test (one-sided) revealed a significantly higher performance (i.e., higher accuracy rate) in the RWM task in the active condition group (*n* = 20) than in the reversed condition group (*n* = 10) (*W* = 62.0, *p*-value = 0.025), but not in the sham condition group (*n* = 10) (*W* = 73.5, *p*-value > 0.05) (**Figure 5B**).

As complementary analyses for the IE score of the three repeated tasks, a two-way ANOVA (3 tasks × 2 tDCS conditions) revealed neither a significant main effect (tDCS condition; *F* = 0.173, *p*-value > 0.05) nor an interaction between the two factors (task × tDCS condition; *F* = 0.501, *p*-value > 0.05). When we divided the control condition into two conditions, i.e., the reversed (*n* = 10) condition and the sham condition (*n* = 10), a two-way ANOVA (3 tasks × 3 tDCS conditions) revealed neither a significant main effect (tDCS condition; *F* = 2.487, *p*-value > 0.05) nor an interaction between the two factors (task × tDCS condition; *F* = 1.691, *p*-value > 0.05).

## Discussion

In the present study, the results of E1 demonstrated that the right LOTC and the FBA are involved in the mental rotation of body part imagery, which supported the previous knowledge regarding LOTC function in the mental rotation task (Tomasino and Gremese, 2015), in the perception of body parts (Downing et al., 2001; Peelen and Downing, 2007; Moro et al., 2008; Bracci et al., 2012; Pitcher et al., 2012), and in their movements and motor representations (Astafiev et al., 2004; Peelen and Downing, 2007; Urgesi et al., 2007a; Ishizu et al., 2009; van Nuenen et al., 2012; Tomasino and Gremese, 2015; Zimmermann et al., 2016). In E2, we tested the hypothesis that active stimulation of the LOTC using tDCS would enhance performance on the mental manipulation in the hand imagery task. The results suggested a possible effect of tDCS on this task. Further study is necessary to clarify whether these results in E1 and E2 were specific for the mental manipulation of body parts but not non-body parts.

Mental rotation is the ability to rotate an object or a body part in one’s mind, i.e., the ability to make perceptual judgments regarding an object’s new spatial configuration in one’s mind. Many neuroimaging studies on the functional neuroanatomy of mental rotation have identified three core brain regions that are considered to serve mental rotation processing: (i) the superior parietal region (Alivisatos and Petrides, 1997; Iwaki et al., 1999; Harris et al., 2000; Richter et al., 2000; Jordan et al., 2001); (ii) the motor and/or premotor regions (Kosslyn et al., 1998, 2001; Vingerhoets et al., 2002); and (iii) the extrastriate visual region (e.g., V5) (Howard et al., 1995; Cohen et al., 1996; Barnes et al., 2000). The neural correlates of the mental rotation of hands in the mind have also been a focus of study (Bonda et al., 1995; Parsons et al., 1995; Kosslyn et al., 1998; Vingerhoets et al., 2002; Wraga et al., 2003; de Lange et al., 2005; Seurinck et al., 2005; de Lange et al., 2006; Creem-Regehr et al., 2007; Corradi-Dell’Acqua et al., 2009; Ferri et al., 2012a; Papeo et al., 2012). One fMRI study reported that the increase in cerebral blood flow (CBF) in the LOTC was greater during the mental rotation of hands than during the mental rotation of objects (Vingerhoets et al., 2002). Coincidentally, the region where the CBF was significantly increased in this study was similar to the area identified using our lesion mapping method, as shown in **Figure 4C**. Our results contribute further evidence that deterioration in the LOTC is associated with lower performance in the mental rotation of body parts. However, using activation likelihood estimation meta-analysis, a recent study reported that the right middle occipital gyrus was activated during the mental rotation of non-bodily stimuli compared to that of bodily related stimuli (Tomasino and Gremese, 2015). This activation was not observed in the reverse contrast (i.e., non-bodily related < bodily related stimuli) (Tomasino and Gremese, 2015). The discrepancy between this meta-analysis and the present study may be explained by two reasons. One possibility is that the LOTC is activated during mental rotation of any targets (not specific for body parts), which was also supported by this previous meta-analysis (Tomasino and Gremese, 2015). The other possibility is that the discrepancy is due to the difference in the imaging methodology. The present study revealed the brain region where functional deterioration induced a disability in mental rotation, but the previous activation likelihood estimation meta-analysis revealed the brain region where the mental rotation task induced cerebral activation. Intriguingly, using transcranial magnetic stimulation with a cortical inhibitory paradigm, recent studies have demonstrated that inhibition of the EBA results in reduced performances in action planning in healthy subjects (Zimmermann et al., 2016) and in patients with Parkinson’s disease (van Nuenen et al., 2012), which is consistent with our results from E1 (i.e., functional deterioration in the LOTC induced the disability in mental rotation).

The exact mechanism by which mental rotation occurs in the human brain is still unclear, but two distinct possible strategies to execute the mental rotation task have been reported (Kosslyn et al., 1998): one is an internal strategy in which one anticipates what he/she would see if one were to physically manipulate the object (or the body part); the other is an external strategy in which one visualizes the consequences of someone else or an external force moving the object (or the body part). The internal strategy may be used in the case of imagery of the hands because the participants were ready to move their hands in their minds. In these cases, the imagery may be focused on the mental motor transformation of the viewer rather than of the viewed object. The external strategy may be used in the case of imagery of objects rotated by an external device such as an electric motor system because this type of stimulus does not prime one to move one’s own hands and therefore does not involve motor processes. Subsequent neuroimaging studies on mental rotation have also supported these two distinct strategies (Kosslyn et al., 2001; Vingerhoets et al., 2002; Zacks, 2008). Although we did not know which strategy our participants preferred when rotating their hands in their mind in this study, both strategies can explain our results. One explanation is that internal hand imagery (possibly governed by the EBA) was necessary for the subjective view (i.e., internal strategy) required to achieve mental rotation of the hands. The other explanation is that objective hand imagery (also possibly governed by the EBA) was required for an external object (i.e., external strategy) to be rotated.

Transcranial direct current stimulation induces a modulatory effect on brain activity (Nitsche and Paulus, 2001), although the participants barely noticed the stimulation. Therefore, tDCS allows for a reliable sham condition (Gandiga et al., 2006). This factor is essential for a double-blind, placebo-controlled study. In the present study (E2), we tested the hypothesized effect of tDCS on the mental manipulation (i.e., mental rotation) of body part imagery. The results showed a significant effect of tDCS on the accuracy ratio of mental manipulation of body part imagery, but not on the IE score. With regard to motor learning, previous studies have demonstrated that anodal stimulation of the primary motor cortex (Nitsche et al., 2003; Antal et al., 2004b), premotor cortex (Wade and Hammond, 2015) and middle temporal V5 (Antal et al., 2004b) resulted in increased performance. In addition, a recent study of human gesture processing showed that anodal stimulation of the left inferior parietal lobe induced better performance (Weiss et al., 2013). Although further studies are necessary to confirm whether anodal stimulation of these brain areas affects the mental manipulation of body part imagery, the right LOTC may be a possible candidate that plays a pivotal role not only in the mental recognition of body parts, as previously reported, but also in the mental manipulation of body part imagery, as shown in the present study.

There were some important limitations of our study. First, we did not employ a mental rotation task with non-body parts (i.e., objects) as a control rotation task in E1 and E2. Therefore, we could not conclude that these results were specific for a mental rotation task with body parts. Second, in E2, the anode electrode was placed over PO8 (international EEG 10/10 system) to activate the LOTC. However, the spatial resolution of tDCS is too low to precisely stimulate functional subdivisions of a cortical area (Woods et al., 2016) and inconsistent behavioral outcomes of tDCS might be caused by individual anatomical differences (Kim et al., 2014). Furthermore, the effect of both cathode and anode stimuli on the right frontal area in the active and in the reversed conditions, respectively, may be a confounding factor in cognitive performance (Seidler et al., 2017). Therefore, we could not exclude the possibility that the effects were driven by cathodal stimulation of the DLPFC rather than anodal stimulation of the LOTC. Further study using simulation methods to estimate brain regional current flow (e.g., the finite element method) is necessary to conclude the facilitative role of the LOTC in the mental rotation task. Third, in E2, we employed healthy subjects but not elderly subjects with dementia. Therefore, we could not test the possibility that tDCS is effective for individuals with cognitive decline in the mental rotation task. Fourth, regarding the IE score in E2, we failed to demonstrated any significant effect of tDCS (i.e., in unpaired *t*-tests and in two-way ANOVA analyses), which may be attributed to the small sample size. Furthermore, these visual tasks might have been too easy for the healthy subjects to accomplish because the difficulty of these tasks targeted elderly subjects in E1 who showed some degree of cognitive decline. Therefore, further improving their IE scores in any of the tasks via anodal stimulation to the LOTC may not have been possible (i.e., a ceiling effect) in the healthy younger subjects. Regarding the accuracy ratio, further improvement may not have been possible in the CRT and WM tasks for the same reason (i.e., a ceiling effect), which also implies that the putative effect of LOTC stimulation in the RWM task might not be specific for the manipulation of body parts. Therefore, further study with a larger sample size and with various visual tasks in which the degree of difficulty is adjusted for the participants is necessary to determine the specific role of the LOTC in the mental rotation task. Despite these limitations, the present results supported the previous knowledge regarding LOTC function in the mental rotation task (Tomasino and Gremese, 2015), in the perception of body parts (Downing et al., 2001; Peelen and Downing, 2007; Moro et al., 2008; Bracci et al., 2012; Pitcher et al., 2012) and in their movements and motor representations (Astafiev et al., 2004; Peelen and Downing, 2007; Urgesi et al., 2007a; Ishizu et al., 2009; van Nuenen et al., 2012; Zimmermann et al., 2016). In addition, our data suggested the facilitative effect of tDCS (anodal stimuli for the right posterior brain area) on performance in the mental rotation task.

## Ethics Statement

This study was approved by the Ethics Committee of Hamamatsu University School of Medicine and Hamamatsu Medical Center and by the Ethics Committee of Kanazawa University Hospital in Japan. Written informed consent was obtained from all participants prior to enrollment. The methods were carried out in accordance with approved guidelines. In case of patients with dementia, written informed consent was obtained from participants and their family prior to enrollment. The methods were carried out in accordance with approved guidelines.

## Author Contributions

All authors listed, have made substantial, direct and intellectual contribution to the work, and approved it for publication.

## Conflict of Interest Statement

The authors declare that the research was conducted in the absence of any commercial or financial relationships that could be construed as a potential conflict of interest.
