# Supplementary material for: The Lateral Occipito-temporal Cortex Is Involved in the Mental Manipulation of Body Part Imagery
Source: Front Hum Neurosci. 2017 Apr 11;11:181. doi: 10.3389/fnhum.2017.00181 (PMC5387072; doi:10.3389/fnhum.2017.00181)
Supplement: Supplementary file 1 [file Data_Sheet_1.docx]

Supplementary Material

**The lateral occipito-temporal cortex is involved in the mental manipulation of body part imagery**

Mitsuru Kikuchi, Tetsuya Takahashi, Tetsu Hirosawa, Yumi Oboshi, Etsuji Yoshikawa, Yoshio Minabe, Yasuomi Ouchi ^*^

*** Correspondence:** Yasuomi Ouchi: ouchi@hama-med.ac.jp

## １．Supplementary Figure

Figure S1

**
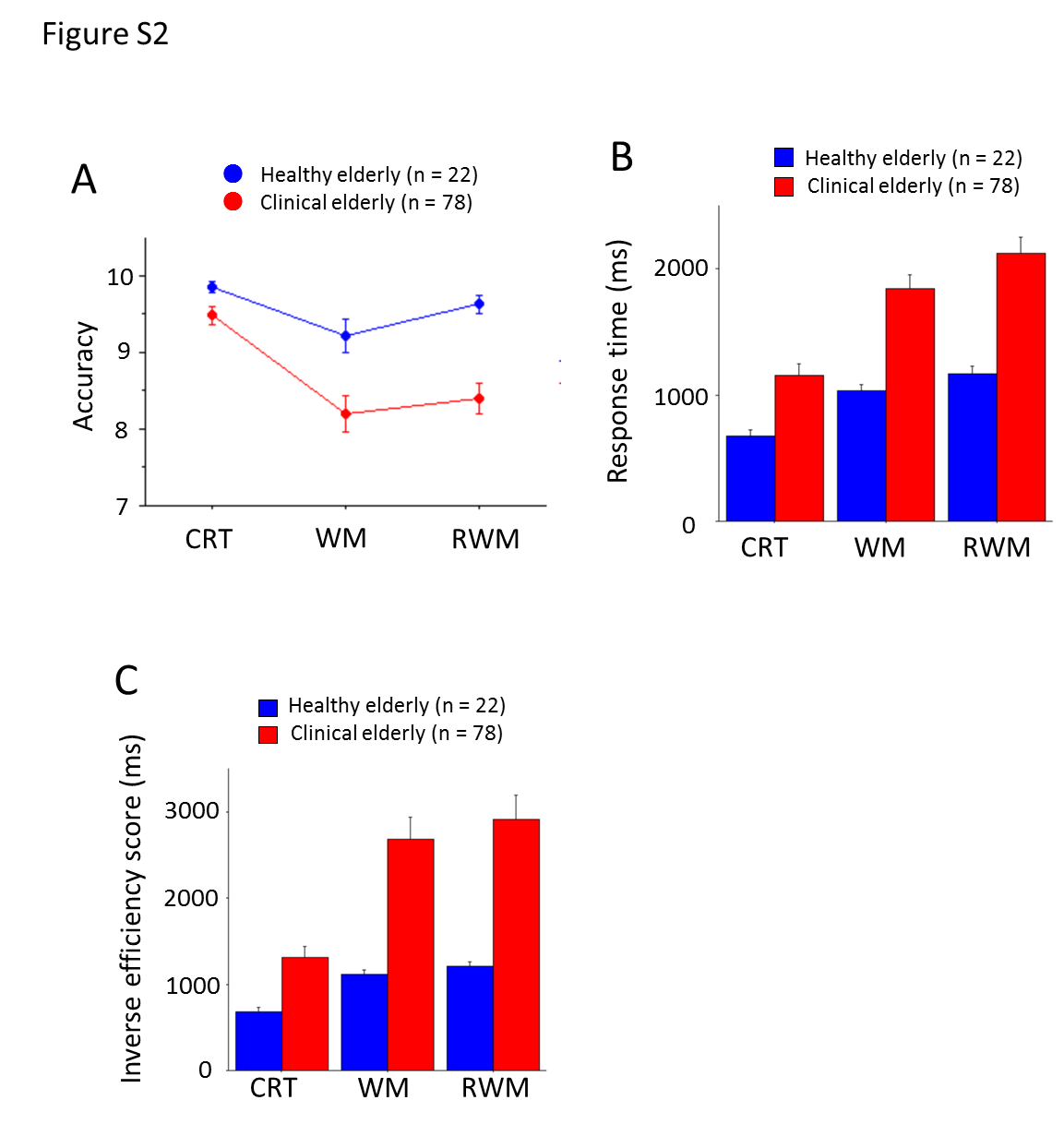
**

**Supplementary Figure S1.** Task performance. (A) Accuracy (maximum of 10 points), (B) response time and (C) inverse efficiency scores for the three tasks (CRT, WM and RWM) in the healthy and clinically diagnosed elderly groups. ms, milliseconds. The error bar indicates 1 standard error.

**Table S1.** Demographic characteristics of all subjects in the tDCS study.

|  | Active group  (i.e., occipital anodal/frontal cathodal)  Mean (SD) | Total control group  (i.e., frontal anodal/occipital cathodal or sham stimuli)  Mean (SD) | Control group (subdivision) | |
| --- | --- | --- | --- | --- |
|  |  |  | Control group 1  (frontal anodal/occipital cathodal)  Mean (SD) | Control group 2  (sham stimuli)  Mean (SD) |
| Total number | 20 | 20 | 10 | 10 |
| Male/Female | 20/0 | 20/0 | 10/0 | 10/0 |
| Age (years) | 23.4(4.5) | 24.8 (6.7) | 25.2 (7.5) | 24.4 (6.2) |
| Education (years) | 15.9 (1.2) | 15.8 (1.4) | 15.7 (1.5) | 15.9 (1.4) |
| Full IQ ^*^ | 108.6 (8.8) | 109.7 (8.3) | 108.7 (8.2) | 110.6 (8.6) |

*The Full IQ score was estimated using the Japanese version of the National Adult Reading Test.
